# Ultrasound findings of the physiological changes and most common breast diseases during pregnancy and lactation*

**DOI:** 10.1590/0100-3984.2015.0076

**Published:** 2016

**Authors:** Antônio Arildo Reginaldo de Holanda, Ana Katherine da Silveira Gonçalves, Robinson Dias de Medeiros, António Manuel Gouveia de Oliveira, Técia Maria de Oliveira Maranhão

**Affiliations:** 1 MSc, Doctoral Student in the Graduate Program in Health Sciences at the Universidade Federal do Rio Grande do Norte (UFRN), Physician at the Maternidade Escola Januário Cicco, Natal, RN, Brazil; 2 PhD, Associate Professor in the Department of Obstetrics and Gynecology at the Universidade Federal do Rio Grande do Norte (UFRN), Natal, RN, Brazil; 3 PhD, Adjunct Professor in the Department of Obstetrics and Gynecology at the Universidade Federal do Rio Grande do Norte (UFRN), Physician at the Maternidade Escola Januário Cicco, Natal, RN, Brazil; 4 PhD, Visiting Professor at the Universidade Federal do Rio Grande do Norte (UFRN), Natal, RN, Brazil; 5 PhD, Full Professor in the Department of Obstetrics and Gynecology at the Universidade Federal do Rio Grande do Norte (UFRN), Natal, RN, Brazil

**Keywords:** Pregnancy, Lactation, Breast, Ultrasonography

## Abstract

Because of the physiological changes that occur during pregnancy and lactation, diagnostic ultrasound of the breast during these periods is a challenge for physicians. Therefore, a comprehensive understanding of imaging, anatomy, and physiology of the breast is important to effectively diagnosing diseases that can arise in women who are pregnancy or lactating. The aim of this article was to review the physiological changes that occur in the breasts during pregnancy and lactation, as well as to describe the main features of the breast diseases that occur most frequently during these periods.

## Abstract (pt)

O diagnóstico ultrassonográfico das mamas durante a gravidez e lactação representa um desafio para o médico, em função das alterações fisiológicas próprias destes períodos. Para tanto, é essencial uma compreensão das imagens, da anatomia e da fisiologia mamárias para diagnosticar mais eficazmente doenças concomitantes. O presente artigo teve como objetivo fazer uma revisão das alterações fisiológicas que ocorrem nas mamas durante a gravidez e lactação, bem como relatar as principais características ultrassonográficas das doenças mamárias mais frequentes nestes períodos.

## INTRODUCTION

Ultrasound evaluation of the breasts during pregnancy and lactation represents a great challenge to physicians, especially because of the various physiological changes, which make the examination more difficult, often preventing or hindering the appropriate interpretation of the findings^(1-3)^. The changes seen on ultrasound imaging of the breast during those periods can simulate the presence of some diseases, as well as making it difficult to assess other, pre-existing, diseases^(4)^. However, some benign processes, such as trauma and inflammation, which can be confused with malignancy, hinder the diagnosis when they occur concomitantly with pregnancy or lactation^5)^. Most breast lesions diagnosed during pregnancy and lactation, even some specific ones such as lactation and adenoma galactocele, are benign^(3)^. The diagnosis of breast cancer, which is difficult to elucidate and is less common among women who are pregnant or lactating than among those of the same age who are not, continues to be a challenge for clinicians^(1)^. In addition, the understanding of the various breast problems and of the characteristics of the corresponding images is essential to establishing an appropriate approach to such patients^(2)^.

The physiological changes during pregnancy and lactation increase breast density^(2,4,6)^, particularly in young women^(4)^, and make it technically difficult to evaluate breast imaging examinations^(4,6)^. Although an increase in breast density limits the use of mammography^(4)^, there is no consensus as to whether the evaluation of the image is so compromised during pregnancy and lactation that the use mammography should be avoided when it is clinically indicated^(7)^. However, because of the risk that ionizing radiation poses to the fetus during the first trimester of pregnancy (i.e., during organogenesis), mammography should be avoided during that period, although it has been shown that there is an association with malformations only when the irradiation is approximately 2 million times higher than the norm^(4)^.

Because normal physiological changes can obscure the diagnosis of breast diseases in pregnant or lactating women, magnetic resonance imaging (MRI) is also not commonly used is such women, ultrasound and mammography being more appropriate^(4)^. However, MRI is indicated in certain situations, such as when cancer occurs during pregnancy and there is a need to assess its extent, as well as to determine whether or not it is multifocal^(4)^. Currently, it is thought that MRI should be used only in certain situations, taking into account the risk-benefit ratio and avoiding gadolinium-based contrast media, although there is no conclusive evidence that the electromagnetic fields generated during the procedure have harmful effects on the fetus^(4)^.

Ultrasound is considered the method of choice during pregnancy and lactation^(2,6)^, with a sensitivity of 86.7% and 100.0%, respectively^(2,4)^, considerably greater than the 30.0% reported for mammography^(2,4,6,8)^. Ultrasound also has the benefits of not exposing the fetus to radiation^(2)^, producing high-resolution images, and allowing a more effective assessment of the breast structures, making it excellent for diagnosing and differentiating between benign and malignant lesions^(1)^. However, whenever an image is suspect, it is necessary to perform mammography^(4)^, biopsy^(9)^, or both.

The use of ultrasound requires a solid knowledge of anatomy and breast diseases, especially when used during lactation^(10)^. Nevertheless, since Cooper studied the lactating breasts by dissection, more than 160 years ago^(10,11)^, there have been few studies investigating the anatomy of the breast during lactation, although inconsistencies in the anatomy of the breast have been observed during and after pregnancy^(11)^.

The aim of this article was to review the physiological changes that occur in the breasts during pregnancy and lactation, as well as to describe the main features of the breast diseases that occur most frequently during these periods.

## PHYSIOLOGICAL CHANGES IN THE BREASTS DURING PREGNANCY AND LACTATION

During pregnancy and lactation, changes in the serum levels of estrogen, progesterone, and prolactin result in physiological changes in the architecture of the breasts^(1,4,12)^, such changes being evident in the histological examination^(4)^.

Under the influence of estrogen, ductal proliferation and growth, as well as, to a lesser degree, alveolar-lobular growth, begin in the first trimester of pregnancy^(1,4,12)^. Expansion of the glandular tissue results in the invasion of adipose tissue, which progresses gradually^(1,4,12)^, occurring simultaneous to increased vascularity and blood flow^(1,4)^.

During the second and third trimesters, progesterone induces lobular hyperplasia, as well as the continuous involution of the fibrofatty stroma^(1,4)^. Although the greatest breast growth occurs up to week 22 of pregnancy, considerable growth can occur in the last trimester and postpartum period in some women^(4)^.

At the end of pregnancy, high levels of estrogens and progesterone counteract prolactin, thus inhibiting milk production^(1,4,13,14)^, although colostrum production occurs in the alveolar cells^(1,13,14)^. The reduction in estrogen and progesterone levels after delivery results in the continuous release of prolactin, caused by stimulation of prolactin-releasing factor in the hypothalamus, and the physical stimulation of the nipple by the newborn promotes the release of oxytocin by the anterior pituitary gland, in order to maintain lactation^(1,13,14)^.

**Figure 1:**
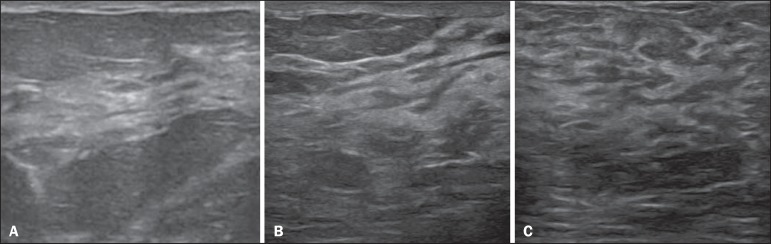
**A:** Breast in the first trimester of pregnancy: predominantly hypoechoic breast parenchyma, showing dilatation of the milk ducts. **B:** Breast in the second trimester of pregnancy: breast parenchyma showing greater echogenicity and more pronounced ductal dilatation than in the first trimester. **C:** Breast in the third trimester of pregnancy: breast parenchyma showing considerably greater echogenicity and extremely more pronounced ductal dilatation in comparison with the second trimester.

**Figure 2:**
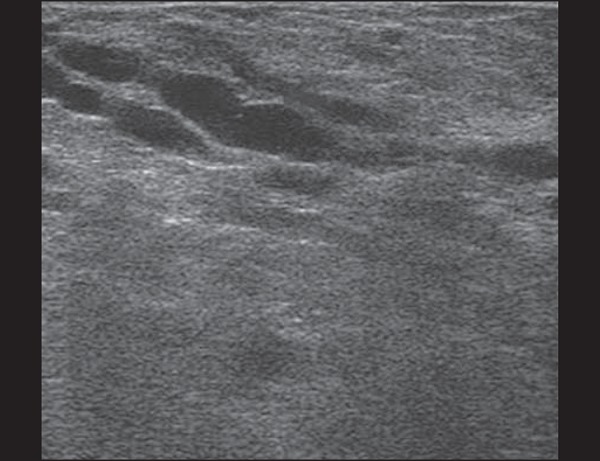
Breast during lactation. Diffusely hyperechoic breast parenchyma, with ductal dilatation due to the accumulation of milk.

This conversion of the breast tissue from a proliferative state during pregnancy to a secretory state during lactation is known as lactogenesis^(4)^. As a result of those changes, the typical image of the breast is diffusely hypoechoic during pregnancy, due to the increase in glandular tissue, becoming diffusely hyperechoic during lactation, as a function of increased vascularity^(1,4)^ and prominence of the ducts^(4)^, as depicted in Figures 1 and 2.

Those physiological changes are manifested clinically by progressive increases in the volume, firmness, and nodularity of the breasts, which makes the clinical examination more difficult^(11)^. In a study evaluating such changes in lactating women^(11)^, the distribution of adipose and glandular tissue was found to vary between women but not between the two breasts of a given woman. The mean number of main ducts observed in that study was 9.6 ± 2.9 for the left breast and 9.2 ± 2.9 for the right breast. The mean diameter of the main ducts, located at the base of the nipple, was 1.9 ± 0.6 mm and 2.1 ± 0.7 mm for the left and right breasts, respectively. The proportion of glandular and adipose tissues was 63 ± 9% and 37 ± 9%, respectively, for the left breast and 65 ± 11% and 35 ± 12%, respectively, for the right breast. However, the authors found that milk production did not correlate with the amount of glandular tissue, the number of ducts, or the mean duct diameter; nor did they find a correlation between the amount of glandular tissue and the storage capacity of the breast^(11)^.

Mammary blood flow, as assessed with pulsed Doppler imaging, has been well studied in animals. However, in humans, the data are still scarce, although it is known that mammary blood flow is primarily increased by the branches of the internal and lateral thoracic arteries, which respectively provide 60-70% and 30% of that flow^(13,14)^. It is believed that the volume of blood flow doubles during pregnancy^(8,15-17)^, concomitant with an increase in metabolic activity and temperature of the breast. The elevated blood flow persists during lactation, appearing to return to pregestational levels two weeks after weaning^(11)^. Some animal studies have shown that there is a positive relationship between milk production and blood flow^(17,18)^ whereas others have found no such relationship^(15,19)^. Among human studies^(15,18)^, there are no reliable data linking increased blood flow during pregnancy with breast milk production^(20)^.

A study involving the use of using color Doppler to evaluate lactating women showed that blood flow varies widely among women, but not between the two breasts of a given woman. Although no relationship has been found between mammary blood flow and milk production, the substantial reduction in mammary blood flow in lactating women with low milk production suggests that there is a blood flow threshold below which milk production is impaired^(15)^.

## INFECTIOUS/INFLAMMATORY CHANGES

### Puerperal mastitis

According to the World Health Organization, mastitis is defined as an inflammatory condition of the breast, with or without infection^(2)^. Breast infections, which rarely occur during pregnancy, are common during breastfeeding^(1,2,4,12)^, with an incidence of 6.6-31.0%^(2,21)^, and the incidence of such infection is highest during the first six weeks after childbirth^(12)^.

Although the causes of breast infections remain obscure^(3)^, the likely etiopathogenic factors include milk stasis, duct obstruction, and breast engorgement, as well as, especially, breast lesion^(2)^, which allows the entry of microorganisms from the nose and mouth of the newborn^(1,4)^ into the breast tissue, through the cracks of the epithelium of the nipple^(1,2,12)^. Incomplete emptying of the breast during breastfeeding predisposes to mastitis^(11)^, because milk is an excellent culture medium, especially when stagnant^(1)^, the most common infectious agents being *Staphylococcus aureus* and *Streptococcus*^(1,4,12)^. Infections with *S. aureus*, which are superficial, present focal invasion from the beginning of the process, whereas those caused by *Streptococcus* are diffuse and cause abscesses only in the advanced stages^(1)^.

**Figure 3:**
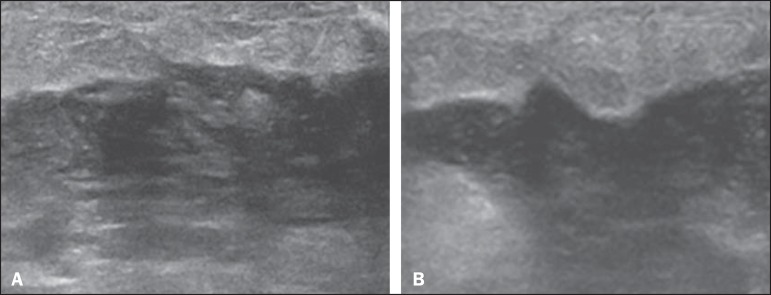
Puerperal mastitis. Amorphous areas of variable echogenicity, predominantly hypoechoic and heterogeneous, diffusely distributed throughout the breast parenchyma, with poorly defined borders.

Clinically, patients with puerperal mastitis present with erythematous and swollen breasts, and clinical suspicion of associated abscess is raised when there is a fluctuant area^(12)^. In a clinical setting, the diagnosis of uncomplicated puerperal mastitis is typically made without difficulty^(3,12)^, there rarely being a need for the use of ultrasound or other imaging methods, although such methods can be indicated, in order to identify abscesses, when the clinical treatment is ineffective^(4,12,20,22)^. However, as can be seen in Figure 3, ultrasound can reveal thickening of the skin, a decrease in the echogenicity of the parenchyma, and increased vascularity (in color Doppler studies), as well as axillary lymph node enlargement^(2)^.

### Abscess

Abscess formation is a common complication of puerperal mastitis^(2,4)^, especially if the treatment has been delayed or inappropriate^(2)^. Among cases of puerperal mastitis, 5-11% evolve to abscess^(2,23)^, the most common infectious agents being *S. aureus* and *Streptococcus*. On clinical examination, patients with puerperal mastitis present with fever, chills, and erythema, as well as the typical signs of mastitis, together with a fluctuant area^(12)^. Ultrasound is the method of choice for diagnosis, as well as for guiding drainage collection and for following the evolution of the condition during the treatment^(1,2)^.

**Figure 4:**
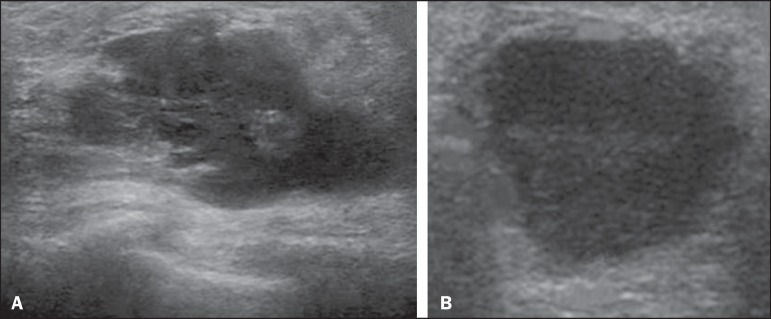
Abscess. **A:** Amorphous, unilocular complex lesion, with ill-defined borders, parallel to the skin, heterogeneous, with variable echogenicity, that is predominantly hypoechoic, with discrete posterior acoustic enhancement, corresponding to an abscess with a fluctuant area. **B:** Ovoid lesion, with well-defined borders, parallel to the skin, hypoechoic, homogeneous, featuring discrete posterior acoustic enhancement, corresponding to abscess formation.

The diagnosis can be more difficult in the presuppurative phase, and mastitis can be confused with a malignant lesion in the suppurative phase^(3)^. Ultrasound typically reveals a complex, hypoechoic formation that varies in shape. The formation is generally multilocular-with ill-defined borders, peripheral vascularization, and posterior acoustic enhancement-and can present central echogenic speckling, which corresponds to degenerative or necrotic tissues^(2,4)^. However, as can be seen in Figure 4, there is no blood supply within the collection^(2,24)^.

In some cases, the appearance of the abscess on ultrasound can suggest other diseases, such as galactocele, which has a highly variable aspect and can present secondary infection, often manifesting as a heterogeneous lesion, with a fat-fluid level^(4)^, which would require ultrasound-guided needle aspiration in order to make the differential diagnosis with abscess^(4)^. If a reliable diagnosis has previously been made by ultrasound, mammography can be indicated and can reveal signs such as masses, distortion, asymmetric density, and thickening of the skin, which are not specific to cancer and call for percutaneous drainage^(2)^.

### Enlargement of intramammary or axillary lymph nodes

**Figure 5:**
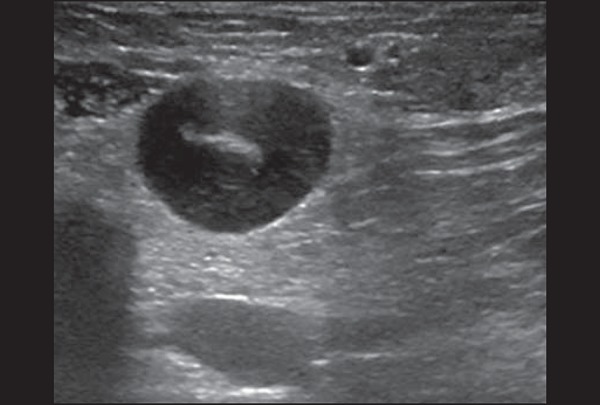
Benign reactive lymph node. Ovoid nodule, parallel to the skin, with well-defined, hypoechoic borders, and a hyperechoic halo, corresponding to a benign lymph node.

In patients with puerperal mastitis, enlarged lymph nodes are usually bilateral and benign, arising in response to inflammation, infectious diseases, neoplasms, or rheumatoid arthritis. Malignant causes include breast cancer metastasis and lymphoma. Enlarged lymph nodes can also arise during lactation, being related to bacterial spread from the nipple during breastfeeding and typically seen in the external upper quadrant of the breast and in the axilla^(2)^. On ultrasound, benign lymph nodes feature hypoechoic borders and hyperechoic halos, whereas hyperplastic lymph nodes typically demonstrate concentric cortical thickening^(2)^, as depicted in Figure 5.

### Granulomatous mastitis

Granulomatous mastitis is a rare, chronic, benign disease of unknown cause^(25)^, associated with pregnancy and lactation, which usually affects young women and can appear months or even years after pregnancy. Although the origin is idiopathic, it has been hypothesized that granulomatous mastitis is caused by *Corynebacterium*^(1,4)^. Another hypothesis is that it is related to an autoimmune reaction to ductal secretion, a reaction in which childbirth, lactation, and the use of oral contraceptives play roles in the development of the disease^(26)^.

It often produces clinical and radiological changes suggestive of inflammatory carcinoma and breast abscess^(1,25,27)^. For that reason and also because it has a tendency to recur and be slow to resolve^(20)^, as well as because lymph node enlargement is seen in 15% of cases^(1,12,27)^, the histopathological diagnosis is indispensable and long-term follow-up is required^(25)^.

The diagnosis is based on exclusion. The histopathological examination usually reveals a granulomatous inflammatory reaction, indicating the need to exclude other diseases, such as tuberculosis, fungal infections, sarcoidosis, Wegener's granulomatosis, as well as the granulomatous reactions found in carcinomas. The imaging findings vary and are sometimes suggestive of malignancy. In women with granulomatous mastitis, mammography might not detect any abnormalities or nonspecific images, such as single or multiple masses, architectural distortion, focal asymmetry, calcifications, and thickening of the skin. It is essential to correlate the imaging with the histopathological examination, with a view toward the possibility of an association with carcinoma, especially when there is no response to corticosteroid therapy^(2)^.

**Figure 6:**
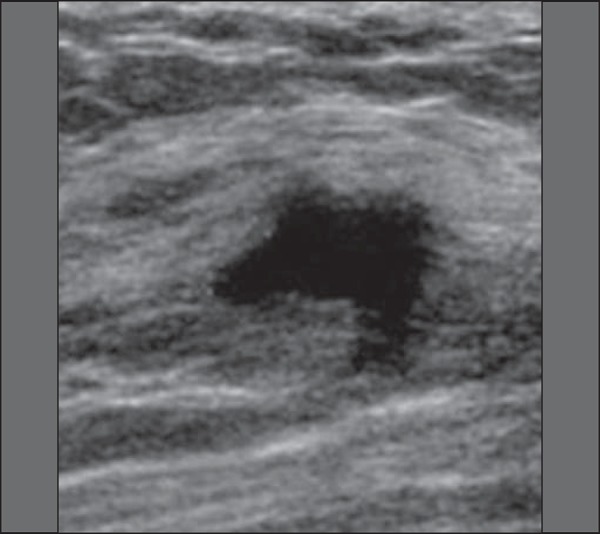
Granulomatous mastitis. Amorphous formation, not parallel to the skin, with ill-defined, hypoechoic borders.

On physical examination and ultrasound, the findings are nonspecific^(20)^, the typical ultrasound presentation being of hypoechoic solitary or multiple nodules or masses^(1,4)^, heterogeneous, with well-defined borders and a tubular aspect. There can also be diffuse abscesses and fistula formation^(2)^, as shown in Figure 6.

## COMMON BENIGN LESIONS

### Galactocele

Galactocele usually occurs as a result of a blocked distal duct, which causes distention of the proximal lobular segments, and presents clinically as a mass that is soft on palpation^(2,4)^ and painless, containing protein, fat, and lactose, and can often present complications such as infection and necrosis^(1,12)^. The most common lesion during lactation^(2,4,11)^, galactocele can be diagnosed in the third trimester of pregnancy or even weeks or months after the cessation of breastfeeding^(1,12)^. In the central portion of the breast, it is often unilocular or bilocular, whereas it is typically multilocular at the periphery^(1)^.

**Figure 7:**
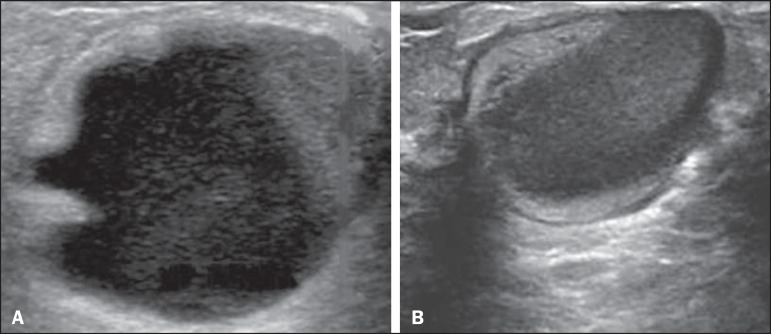
Galactocele. **A:** Lesion, parallel to the skin, with well-defined borders, showing anechoic (cystic) and echogenic (solid) components, with discrete posterior acoustic enhancement and well-defined borders. **B:** Predominantly hypoechoic lesion, parallel to the skin, with welldefined borders, peripheral areas of hyperechogenicity, and posterior acoustic enhancement.

On ultrasound, galactocele has a variable aspect, depending on the amount of fat, protein, and water it contains. The classic aspect is that of a cystic lesion with posterior acoustic shadowing, with thin or coarse speckling corresponding to fat particles in suspension^(2,20)^, as depicted in Figure 7. It can present as single or multiple lesions^(2,4)^, which can be unilateral or bilateral^(4)^, or as a cystic lesion with well-defined borders, consistent with a benign process^(2,4)^. It can also present characteristics of a malignant mass, including an irregular shape and ill-defined borders^(4)^. The interior of the galactocele varies from homogeneous to heterogeneous, by the presence of echoes, depending on the content^(2)^.

Mammography and MRI can be required when there is suspicion of diseases such as cancer and abscess, because the demonstration of fat or a fat-fluid level by those methods can confirm the diagnosis. If the results are inconclusive, aspiration can be recommended as a diagnostic and therapeutic measure^(4)^.

### Lactating adenoma

Lactating adenoma is a benign tumor caused by physiological changes, especially those occurring during lactation and in the third trimester of pregnancy^(1,4,12)^, although it can also arise in the first or second trimester^(11)^. It is sometimes interpreted as a variant of fibroadenoma, tubular adenoma, or lobular hyperplasia, which are also caused by physiological changes^(1,28,29)^.

It is the most common tumor during pregnancy^(2)^, evolves to volume reduction, and can resolve spontaneously in the third trimester^(2,4)^ or during lactation^(1,2,4,12)^, sometimes also due to necrosis^(1,30)^.

Clinically, lactating adenoma manifests as a palpable mass^(1,2,12)^, described as painless, soft, and mobile^(12)^, which can occasionally recur in subsequent pregnancies^(1,2)^, although recurrence is unusual after complete surgical resection^(1)^. When infarction occurs, the adenoma can become clinically atypical, manifesting as a firm mass^(12)^.

**Figure 8:**
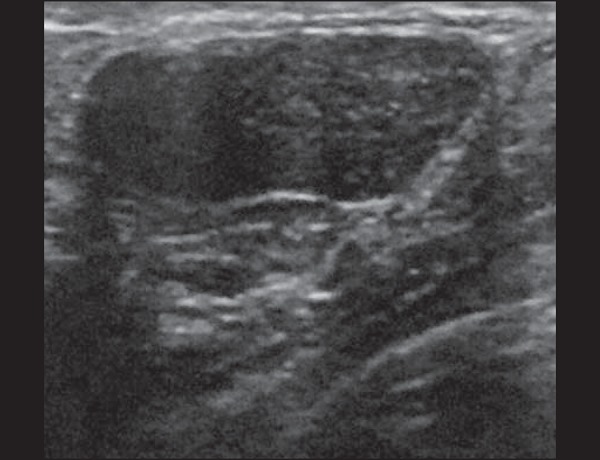
Lactating adenoma. Ovoid nodule, parallel to the skin, with a heterogeneous, hypoechoic pattern and well-defined borders.

On ultrasound, it is difficult to distinguish between lactating adenoma and fibroadenoma^(1,4,12)^. Ultrasound findings are usually consistent with a benign process but are nonspecific^(7)^, including low echogenicity^(4)^. Lactating adenoma most often manifests as an oval mass, the longest axis being parallel to the skin, with well-defined borders, a homogenous texture, and posterior acoustic shadowing. It can still have discrete lobulation, which presents poorly defined boundaries with the surrounding tissue^(1)^, as well as showing discrete blood flow in color Doppler studies^2)^. Like fibroadenoma, lactating adenoma can manifest as multiple, bilateral lesions^(12)^. The differential diagnosis with malignant lesions can be difficult to make when there is infarction^(4,28,30,31)^ or necrosis^(1)^, due to the ill-defined borders^(1,12)^, peripheral microlobulation^(1)^, structural heterogeneity, and posterior acoustic shadowing^(1,4,13)^, as shown in Figure 8.

### Fibroadenoma

Fibroadenoma is common in young, non-pregnant women, often increasing volume during pregnancy and lactation^(1,2)^, in response to rising estrogen levels^(1,4)^. Like lactating adenoma, fibroadenoma typically regresses after the cessation of breastfeeding^(4)^.

On ultrasound, fibroadenoma is usually indistinguishable from lactating adenoma^(1,4)^, being predominantly hypoechoic^(2,4)^, round or oval in shape, with a homogeneous texture, well-defined borders, a pseudocapsule, no posterior acoustic shadowing, and normal adjacent tissue^(2)^. However, during pregnancy, fibroadenoma can have an atypical cystic appearance, increased vascularization, and prominent ducts^(2)^.

**Figure 9:**
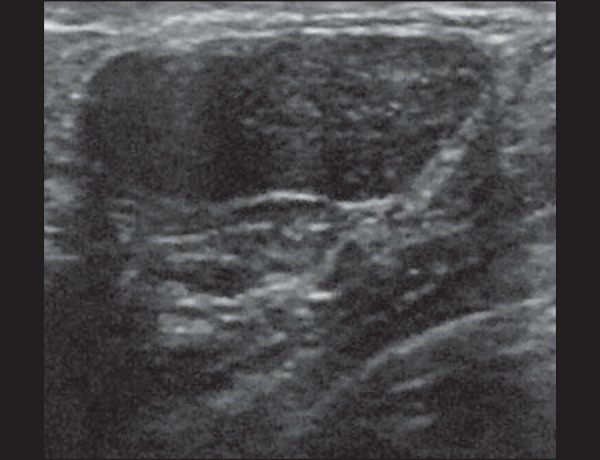
Fibroadenoma. Ovoid nodule, parallel to the skin, with a homogeneous, hypoechoic pattern, well-defined borders, and discrete posterior acoustic enhancement.

As in lactating adenoma, infarction can occur, due to the relative decrease in vascular supply, appearing more heterogeneous on ultrasound^(4,18,28,30)^. The presence of atypical features, such as microlobulation, ill-defined borders, a heterogeneous echotexture, posterior acoustic shadowing, and pronounced hypoechogenicity (Figure 9), can indicate the need for percutaneous biopsy in order to confirm the diagnosis^(2)^.

## PREGNANCY-ASSOCIATED BREAST CANCER

Pregnancy-associated breast cancer is defined as that which occurs concomitantly with pregnancy or up to one year after childbirth. It accounts for 3% of all cases of breast cancer, with an incidence of one case in every 3,000-10,000 pregnancies^(1,2)^, the current tendency being toward an increase, due to the increasing number of women who conceive later in life^(4)^. In general, pregnancy-associated breast cancer is biologically aggressive; estrogen- and progesterone-receptor negative; and positive for human epidermal growth factor receptor type 2^(2)^.

In view of its high sensitivity, together with its ability to assess the axillary lymph nodes and monitor the response to chemotherapy, ultrasound is the ideal method to detect a latent image during pregnancy. However, if a suspicious lesion is observed on ultrasound, mammography, which is considered a safe method, should be performed^(4,20,31-33)^, as should ultrasound-guided biopsy^(34)^. In mammography and ultrasound, the imaging rarely differs significantly from that of cancer in non-pregnant women^(2,4)^. If the lesion is considered highly suspect or if the biopsy is positive, the ipsilateral axilla should also be assessed^(34)^.

In comparison with cancer in non-pregnant women of the same age, pregnancy-associated breast cancer tends to produce a larger tumor, is diagnosed later, and presents a worse prognosis^(4,34)^. Patients with pregnancy-associated breast cancer typically present with a palpable, painless mass^(4)^, attached to the deep planes^(5)^, with diffuse edema and erythema in the early phases of the disease^(4)^.

**Figure 10:**
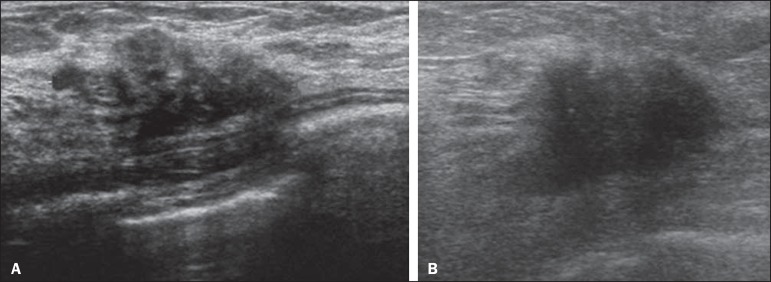
Pregnancy-associated breast cancer. **A:** Amorphous formation, parallel to the skin, with variable echogenicity (predominantly hypoechoic), a heterogeneous texture, discrete acoustic enhancement and poorly defined borders. **B:** Irregular, hypoechoic, heterogeneous nodule, parallel to the skin, featuring discrete acoustic shadowing and poorly defined borders.

On ultrasound, the mass appears heterogeneous (hypoechoic or complex), with a transverse diameter equal to or less than its vertical diameter (not parallel to the skin), an irregular shape, ill-defined borders, a variable echogenic halo, and posterior acoustic shadowing^(2,5)^. In situ ductal carcinoma, which is associated with microcalcifications, is easily detected on mammography and is often not observed on ultrasound^(5)^. Other findings include thickening of the suspensory ligaments of the breast, skin edema, and enlargement of the axillary lymph nodes^(2)^, as depicted in Figure 10. In color Doppler studies, the pattern of vascularization is chaotic. Some carcinomas are quite subtle, with echogenicity similar to that of the surrounding tissues^(5)^.

## CONCLUSION

The ultrasound diagnosis of breast diseases during pregnancy and lactation is challenging because of the hormonal changes characteristic of those periods, which can modify the appearance of the image. Depending on the nature of the suspected diagnosis, other methods of imaging or biopsy might be needed in order to elucidate the diagnosis. Doing so requires an adequate understanding of the physiological changes and benign mammary lesions that commonly occur during those periods, in order to differentiate between such lesions and pregnancy-associated breast cancer. Thus, a delay in diagnosis can be avoided, allowing a satisfactory approach and more effective treatment.

## References

[1] Yu JH, Kim MJ, Cho H (2013). Breast diseases during pregnancy and lactation. Obstet Gynecol Sci.

[2] Joshi S, Dialani V, Marotti J (2013). Breast disease in the pregnant and lactating patient:
radiological-pathological correlation. Insights Imaging.

[3] Boisserie-Lacroix M, Dos Santos E, Belléannée G (2004). La femme enceinte: difficultés
diagnostiques. Imagerie de la Femme.

[4] Canoy JM, Mitchell GS, Unold D (2012). A radiologic review of common breast disorders in pregnancy and
the perinatal period. Semin Ultrasound CT MR.

[5] Svensson WE (1997). A review of the current status of breast
ultrasound. Eur J Ultrasound.

[6] Bock K, Hadji P, Ramaswamy A (2006). Rationale for a diagnostic chain in gestational breast tumor
diagnosis. Arch Gynecol Obstet.

[7] Swinford AE, Adler DD, Garver KA (1998). Mammographic appearance of the breasts during pregnancy and
lactation: false assumptions. Acad Radiol.

[8] Lee SS, Hartman HJ, Kuzmiak CM (2013). Management of breast symptoms in the pregnant and lactating
patient. Curr Obstet Gynecol Rep.

[9] Sumkin JH, Perrone AM, Harris KM (1998). Lactating adenoma: US features and literature
review. Radiology.

[10] Geddes DT (2007). Inside the lactating breast: the latest anatomy
research. J Midwifery Womens Health.

[11] Ramsay DT, Kent JC, Hartmann RA (2005). Anatomy of the lactating human breast redefined with ultrasound
imaging. J Anat.

[12] Vashi R, Hooley R, Butler R (2013). Breast imaging of the pregnant and lactating patient: physiologic
changes and common benign entities. AJR Am J Roentgenol.

[13] Rosen PP, Rosen PP (2001). Anatomic and physiologic morphology. Rosen's breast pathology.

[14] Neville MC (2001). Anatomy and physiology of lactation. Pediatr Clin North Am.

[15] Gedds DT, Aljazaf KM, Kent JC (2012). Blood flow characteristics of the human lactating
breast. J Hum Lact.

[16] Vorherr H (1974). The breast: morphology, physiology and lactation.

[17] Thoresen M, Wesche J (1988). Doppler measurements of changes in human mammary and uterine
blood flow during pregnancy and lactation. Acta Obstet Gynecol Scand.

[18] Stelwagen K, Davis SR, Farr VC (1994). Mammary epithelial cell tight junction integrity and mammary
blood flow during an extended milking interval in goats. J Dairy Sci.

[19] Lacasse P, Prosser CG (2003). Mammary blood flow does not limit milk yield in lactating
goats. J Dairy Sci.

[20] Sebate JM, Clotet M, Torrubia S (2007). Radiologic evaluation of breast disorders related to pregnancy
and lactation. Radiographics.

[21] Kvist LJ, Larsson BW, Hall-Lord ML (2008). The role of bacteria in lactational mastitis and some
considerations of the use of antibiotic treatment. Int Breastfeed J.

[22] Marchant DJ (2002). Inflammation of the breast. Obstet Gynecol Clin North Am.

[23] Ulitzsch D, Nyman MK, Carlson RA (2004). Breast abscess in lactating women: US-guided
treatment. Radiology.

[24] Karstrup S, Solvig J, Nolsoe CP (1993). Acute puerperal breast abscesses: US-guided
drainage. Radiology.

[25] Goulart APS, Silva RS, Volbrecht B (2011). Mastite granulomatosa lobular idiopática: relato de
caso. Rev Bras Mastologia.

[26] Hur SM, Cho DH, Lee SK (2013). Experience of treatment of patients with granulomatous lobular
mastitis. J Korean Surg Soc.

[27] Han BK, Choe YH, Park JM (1999). Granulomatous mastitis: mammographic and sonographic
appearances. AJR Am J Roentgenol.

[28] Baker TP, Lenert JT, Parker J (2001). Lactating adenoma: a diagnosis of exclusion. Breast J.

[29] Saglam A, Can B (2005). Coexistence of lactating adenoma and invasive ductal
adenocarcinoma of the breast in a pregnant woman. J Clin Pathol.

[30] Behrndt VS, Barbakoff D, Askin FB (1999). Infarcted lactating adenoma presenting as a rapidly enlarging
breast mass. AJR Am J Roentgenol.

[31] Son EJ, Oh KK, Kim EK (2006). Pregnancy-associated breast disease: radiologic features and
diagnostic dilemmas. Yonsei Med J.

[32] Hogge JP, De Paredes ES, Magnant CM (1999). Imaging and management of breast masses during pregnancy and
lactation. Breast J.

[33] Taylor D, Lazberger J, Ives A (2011). Reducing delay in the diagnosis of pregnancy-associated breast
cancer: how imaging can help us. J Med Imaging Radiat Oncol.

[34] Vashi R, Hooley R, Butler R (2013). Breast imaging of the pregnant and lactating patient: imaging
modalities and pregnancy-associated breast cancer. AJR Am J Roentgenol.

